# The role of measurement in moral injury care: latent profiles on the moral injury and distress scale

**DOI:** 10.3389/fpsyt.2025.1691018

**Published:** 2026-01-19

**Authors:** Brandon J. Griffin, Elise A. Warner, Matthew L. McCue, Robert H. Pietrzak, Carmen P. McLean, Sonya B. Norman, Shira Maguen

**Affiliations:** 1 Mental Health Service and Center for Mental Health Care and Outcomes Research, Central Arkansas Veterans Healthcare System, Little Rock, AR, United States; 2 Psychiatric Research Institute, University of Arkansas for Medical Sciences, Little Rock, AR, United States; 3 Center for Excellence in Stress and Mental Health, San Diego VA Health Care System, San Diego, CA, United States; 4 Psychiatry Department, University of California San Diego School of Medicine, San Diego, CA, United States; 5 Clinical Neurosciences Division, National Center for Posttraumatic Stress Disorder (PTSD), West Haven, CT, United States; 6 Department of Psychiatry, Yale School of Medicine, New Haven, CT, United States; 7 Dissemination and Training Division, National Center for Posttraumatic Stress Disorder, Menlo Park, CA, United States; 8 Department of Psychiatry and Behavioral Sciences, Stanford University School of Medicine, Stanford, CA, United States; 9 Executive Division, National Center for Posttraumatic Stress Disorder (PTSD), White River Junction, VT, United States; 10 Mental Health Service and Center for Data to Discovery and Delivery Innovation (3DI), San Francisco VA Health Care System, San Francisco, CA, United States; 11 Department of Psychiatry and Behavioral Sciences, University of California San Francisco School of Medicine, San Francisco, CA, United States

**Keywords:** depression, latent profile analysis, measurement, moral injury, moral injury and distress scale, posttraumatic stress

## Abstract

**Introduction:**

Although studies of moral injury proliferated over the past decade, few studies have examined common moral injury symptom presentations.

**Methods:**

Data were analyzed from a population-based sample (*N* = 645) of combat veterans, healthcare workers, and first responders. All participants endorsed exposure to a potentially morally injurious event (PMIE) and completed the Moral Injury and Distress Scale (MIDS).

**Results:**

Latent profile analysis revealed three distinct symptom profiles based on MIDS items assessing psychological, emotional, social, and spiritual symptoms of moral injury. The majority of participants (74.7%, *n* = 482) reported minimal moral injury symptoms. Approximately one in five participants (20.2%, *n* = 130) endorsed moderate levels of moral injury symptoms, and one in twenty (5.1%, *n* = 33) reported severe moral injury symptoms. All participants with the severe profile screened positive for probable moral injury on the MIDS (score ≥ 27); however, 9.1% had scores within normal limits on measures of posttraumatic stress and depression. After adjusting for demographics, those with moderate or severe moral injury symptoms were more likely than those with minimal moral injury symptoms to be younger, have fewer years of education, and identify as Hispanic.

**Discussion:**

These findings support the use of the MIDS in measurement-based care to identify distinct clinical presentations of moral injury, including those with moderate to severe presentations that warrant further evaluation and potentially treatment.

## Introduction

Individuals internalize beliefs about morally acceptable conduct from various sources, including culture, occupational norms, family values, social groups, and personal experiences (1). Unfortunately, those employed in high stress, service-oriented occupations may be exposed to events in their workplaces that violate these beliefs. For example, military veterans might participate in combat operations involving civilian casualties, and healthcare workers may limit access to potentially life-saving treatments when they triage patients during mass-casualty events. Even when consistent with the relevant ethical standards (e.g., military rules of engagement, medical codes of ethics), they may perceive their own or others’ conduct as violations of their internalized moral beliefs (e.g., care for vulnerable others). In these cases, if they believe that they participated in an event that violated their beliefs because of what they did or failed to do, or if they witness or learn about wrongs committed by others in a group or institution to which they belong, they may sustain a moral injury. Symptoms of moral injury include but are not limited to intractable moral emotions (e.g., guilt and shame), withdrawal from close relationships or valued groups, and loss of meaning or purpose in life or work (2–4).

In a recent population-based survey of military veterans, healthcare workers, and first responders (*N* = 1232), nearly half (47.3%) reported exposure to at least one potentially morally injurious event (5). Additionally, about one in twenty individuals (6.0%) screened positive for probable moral injury, indicating the presence of moral injury symptoms severe enough to negatively impact their ability to function in daily life. In another nationally representative study of U.S. combat veterans, 5.9% screened positive for moral injury, with an additional 4.1% reporting subclinical levels of moral distress (6). Exposure to potentially morally injurious events and moral injury are both linked to elevated rates of psychiatric symptoms, with posttraumatic stress and depression being the most commonly examined (7–10). Similarly, moral injury is associated with increased risk of suicidal thoughts and behaviors, even after accounting for psychiatric comorbidities (11, 12).

## Conceptualization and measurement of moral injury

Like other stress-related syndromes elicited by exposure to a traumatic event (e.g., posttraumatic stress disorder [PTSD]), moral injury stems from exposure to a highly stressful and morally salient event, referred to as a potentially morally injurious event (PMIE). In fact, most studies on moral injury conducted to date employ questionnaires that assess the extent to which respondents endorse being exposed to PMIEs, such as the Moral Injury Events Scale (MIES), rather than symptoms related to the psychological, emotional, social, and spiritual sequelae of exposure (i.e., moral injury (13, 14). Although information obtained from measures of exposure may facilitate case conceptualization and treatment planning, these questionnaires are not efficient screeners for identifying those in greatest need of care because most individuals exposed to a PMIE will not experience clinically significant problems. Rather, moral distress occurs on a spectrum. It can be acute and adaptive when it motivates reparation or advocacy, but at more severe levels, it can be chronic and disabling (15–17). For this reason, recently published questionnaires that screen for the psychological, emotional, social, and spiritual symptoms of moral injury rather than mere PMIE exposure are the optimal measurement tools to identify individuals likely to benefit from targeted treatment (for reviews, see (7, 18)). In the current study, we utilized one of these measures, the Moral Injury and Distress Scale (MIDS) (19).

The MIDS is a valuable tool to screen for moral injury for several reasons (19, 20). First, the MIDS anchors reported distress to a specific PMIE exposure by including an open-response item for respondents to describe the event, alongside forced-choice items that assess whether the exposure occurred because of something the respondent did, failed to do, or witnessed. This helps to eliminate ambiguity about the source of the respondent’s distress, ensuring that moral distress is distinguished from general psychological distress. Second, the MIDS is easy to use for patients and clinicians. Similar to other measures of mental health symptom severity, patients rate the extent to which they were bothered by an array of potential problems in the past month from “not at all” to “extremely.” Because the MIDS is a unidimensional tool, clinicians may sum these responses into a continuous total score, with higher values indicating greater symptom severity. Scores equal to or greater than 27 also indicate probable moral injury, signifying a need for further evaluation and potentially treatment (20). Finally, the MIDS was developed and validated using a diverse population-based sample of U.S. military veterans, healthcare workers, and first responders. In contrast, most other measures were developed solely with military veterans (7), potentially excluding civilian populations who may also be exposed to PMIEs and experience moral injury.

## The current study

The past decade has seen a surge in studies of moral injury. Specifically, the recent development of questionnaires to assess the psychological, emotional, social, and spiritual features of moral injury is a notable improvement upon prior work that assessed PMIE exposure but not the impacts of exposure (19, 21). A next step in this program of research is to empirically test for distinct symptom presentations based on these questionnaires. Doing so could enable clinicians and researchers to better identify those at risk of adverse outcomes due to moral injury and tailor interventions to develop personalized treatment plans. To that end, the objectives of this study were to: 1) identify groups of participants characterized by distinct profiles of responding to the MIDS items; 2) compare mental health symptoms and psychosocial functioning across the identified groups; and 3) examine the extent to which membership in the observed profiles differs by sex, age, race, ethnicity, education level, and religious affiliation.

## Materials and methods

### Participants and procedures

Participants in the parent study (*N* = 1,232) were combat veterans, healthcare workers, and first responders, drawn from KnowledgePanel, an online survey panel with a nationally representative sample of over 50,000 households (19). For military veterans, combat status was determined by providing affirmative responses to two prescreening questions about prior military service and deployment to a combat zone. Healthcare workers and first responders were identified as having worked in their field for at least six months and either still work in or have retired no more than five years prior. **Table 1** displays sociodemographic characteristics.

**Table 1:** Sample characteristics for full sample and by profile.

Outcome variable	Full sample (*n* = 645)	Minimal moral injury (*n* = 482)	Moderate moral injury (*n* = 130)	Severe moral injury (*n* = 33)
Sex				
Male	381 (59.1%)	289 (60.0%)	72 (55.4%)	20 (60.6%)
Female	264 (40.9%)	193 (40.0%)	58 (44.6%)	13 (39.4%)
Age (years)				
18-39	127 (19.7%)	89 (18.5%)	29 (22.3%)	9 (27.3%)
40-59	218 (33.8%)	154 (32.0%)	46 (35.4%)	18 (54.5%)
60+	300 (46.5%)	239 (49.6%)	55 (42.3%)	6 (18.2%)
Race and ethnicity				
White, Non-Hispanic	500 (77.5%)	390 (80.9%)	92 (70.8%)	18 (54.5%)
Black, Non-Hispanic	47 (7.3%)	38 (7.9%)	6 (4.6%)	3 (9.1%)
Other, Non-Hispanic	13 (2.0%)	5 (1.0%)	7 (5.4%)	1 (3.0%)
Hispanic	62 (9.6%)	33 (6.8%)	20 (15.4%)	9 (27.3%)
2+ Races, Non-Hispanic	23 (3.6%)	16 (3.3%)	5 (3.8%)	2 (6.1%)
Education				
No high school diploma/GED	1 (0.2%)	0 (0.0%)	1 (0.8%)	0 (0.0%)
High school	39 (6.0%)	30 (6.2%)	8 (6.2%)	1 (3.0%)
Some college or Associates degree	248 (38.4%)	177 (36.7%)	58 (44.6%)	13 (39.4%)
Bachelor’s degree	199 (30.9%)	147 (30.5%)	40 (30.8%)	12 (36.4%)
Master’s degree or above	158 (24.5%)	128 (26.6%)	23 (17.7%)	7 (21.2%)
Religion				
Catholic	154 (23.9%)	112 (23.2%)	30 (23.1%)	12 (36.4%)
Protestant	242 (37.5%)	190 (39.4%)	42 (32.3%)	10 (30.3%)
Other Christian	64 (9.9%)	50 (39.4%)	13 (10.0%)	1 (3.0%)
Other not Christian	38 (5.9%)	25 (5.2%)	10 (7.7%)	3 (9.1%)
Spiritual/nonreligious	66 (10.2%)	49 (10.2%)	14 (10.8%)	3 (9.1%)
Atheist/Agnostic	79 (12.2%)	54 (11.2%)	21 (16.2%)	4 (12.1%)

Because the purpose of the parent study was to recruit a nationally representative sample of individuals including those with and without PMIE exposure and PMIE exposure is a prerequisite of moral injury, we added an additional inclusion criterion for this secondary analysis focused on moral injury symptoms. Specifically, participants were asked if they have been exposed to a PMIE either by participating in an event because of what they did (i.e., commission), what they failed to do (i.e., omission), or by witnessing others’ wrongful acts. Two authors (S.M., S.B.N.) reviewed the descriptions prior to statistical analysis; 279 cases were considered invalid for providing insufficient information, which made it unclear whether they had experienced a PMIE or not (e.g., “I wish I knew the things I now know”) or for giving an example that did not describe a PMIE (e.g., “Personal goals that I did not achieve”). The final sample included only respondents who endorsed exposure to a valid PMIE (*n* = 645). Those who denied PMIE exposure or provided insufficient information to validate the exposure were excluded from the current study.

### Measures

Participants completed the MIDS alongside mental health symptom measures for posttraumatic stress, depression, and psychosocial functioning.

#### Moral injury and distress scale

The MIDS is a comprehensive measure of PMIE exposure and outcomes (19). The measure consists of two parts. Part 1 includes 6 items which assess the following three types of PMIE exposure: 1) participating in a PMIE by commission, 2) participating in a PMIE by omission, and 3) witnessing a PMIE. Participants were asked about the extent to which they were exposed to and bothered by each type of PMIE exposure using a 5-point Likert scale from 0 (*not at all*) to 4 (*extremely*). If respondents endorsed any MIDS Part 1 item, they were prompted to complete the MIDS Part 2 items. The MIDS Part 2 includes 18 items that assess how bothered the respondent was in the past month by possible cognitive, emotional, social, and spiritual reactions indexed to a specific PMIE. Example items include “I think about how I should have been able to do more” and “I don’t seek support because I worry others would not understand.” They responded to each item using a 5-point response format (0 = *not at all*, 4 = *extremely*). We aggregated item responses into a sum score, with higher scores indicating greater severity of moral distress, and we used the recommended cut score to identify probable moral injury (score = 27; 20). In the current sample, internal consistency of scores on the MIDS Part 2 items was excellent (α = .93).

#### Posttraumatic stress symptoms

The PTSD Checklist for DSM-5 (PCL-5; (22) is a 20-item self-report measure of severity of PTSD symptoms, based on criteria for PTSD outlined in the 5^th^ edition of the Diagnostic and Statistical Manual of Mental Disorders (23). The questionnaire asks how bothered a person was by each potential symptom over the past month. Scores are on a Likert scale of 0 (*not at all*) to 4 (*extremely*). Responses were summed, with possible total scores ranging from 0-80. Higher scores indicated more severe PTSD symptoms. A clinical cut-off score of 33 indicated probable PTSD (24). In the current sample, internal consistency of scores on the PCL-5 items was excellent (α = .97).

#### Depressive symptoms

The Patient Health Questionnaire-9 (PHQ-9; (25) is a nine-item measure of major depressive symptoms, which were based on criteria for major depressive disorder as outlined in the DSM-IV (26). Participants were asked about how frequently they experienced each depressive symptom over the past 2 weeks, with responses on a Likert scale from 0 (*not at all)* to 3 (*nearly every day)*. Responses were summed, with possible total scores ranging from 0-27. Higher scores indicated more severe depressive symptoms. Consistent with prior literature, we used a cut-off score of 10 to indicate positive screen for a probable depressive disorder (25). In the current sample, internal consistency of scores on the PHQ-9 items was excellent (α = .92).

#### Psychosocial functioning

The Brief Inventory of Psychosocial Functioning (B-IPF; (27) is an abridged version of the 80-item Inventory of Psychosocial Functioning (28). The B-IPF is a 7-item measure of PTSD-related functional impact endorsed across seven domains (romantic relationships, relationships with children, family relationships, friendships, work, training/education, and daily activities). Each item represents one of the functional domains. Responses are on a 6-point Likert scale from 0 (*never*) to 6 (*always*). A total score is calculated by summing items, dividing the total score by the maximum possible score based on number of items scored, and multiplying by 100. Higher scores indicate greater functional impairment, and scores equal to or greater than 31 indicate at least a moderate level of impairment (27). In the current sample, internal consistency of scores on the B-IPF items was excellent (α = .88).

### Data analysis

We conducted a latent profile analysis (LPA) to identify latent groups distinguished by unique response patterns to the 18 MIDS Part Two items assessing symptoms of moral injury. Using Mplus version 8.4 (29), we specified a series of models wherein items assessing psychological, emotional, social, and spiritual symptoms of moral injury were regressed onto a discrete latent variable representing the number of unique response patterns (i.e., profiles). The number of profiles specified for the discrete latent variable was increased iteratively in successive models. To determine the model with the optimal number of profiles, we evaluated multiple fit indices, including the Lo-Mendell-Rubin Likelihood Ratio Test (LMR) and Bootstrapped Likelihood Ratio Test (BLRT). Statistically significant *p* values for the LMR and BLRT suggested that the specified model fit the data better than the model with one less profile (30). Lower values for the Akaike Information Criterion (AIC), Bayesian Information Criteria (BIC), and sample size adjusted Bayesian Information Criteria (aBIC) also indicated better fit (31). Additionally, we considered entropy values and average posterior probabilities (APPs) to evaluate the quality of fit across models, with values closer to 1 and no less than .70 suggesting greater accuracy of classification (32). Finally, models containing profiles to which more than 5% of participants were assigned and models with profiles that consistently emerged across solutions were prioritized over models containing profiles with fewer participants and exhibiting less stability across solutions (33).

To validate the identified profiles by examining between group differences in mental health symptom severity, we tested for equality of mean scores on measures of posttraumatic stress, depression, and psychosocial functioning between the latent profiles using Wald chi-square tests, as recommended by Asparouhov and Muthén in their three-step approach for comparing distal outcomes in mixture models (34). We also examined factors associated with likelihood of assignment to the observed profiles by estimating a multivariable binary logistic regression. In these models, we specified profile assignment as the dependent variable, with assignment to the modal profile (minimal symptoms) as the reference category compared to all other profiles. For the regression model, we planned to aggregate symptomatic profiles for this analysis to reduce the likelihood of biased model parameters for profiles containing a small percentage of the overall sample. Independent variables, including demographic characteristics, were simultaneously entered into the model. These characteristics included sex, age, race and ethnicity, highest educational attainment, and religious affiliation. Levels of independent variables that included less than 10% of the sample were collapsed to facilitate interpretation.

## Results

### Model selection

**Table 2** displays fit indices for models specifying one to five profiles. We evaluated the fit of each solution, from the most to least complex model, to identify the solution with the fewest number of profiles and best overall fit. The five- and four-profile models were not ideal because less than 5.0% of the sample was assigned to one or more profiles. The LMR also indicated that the five-profile solution did not fit the data better than the four-profile solution, and the four-profile solution did not fit the data better than the three-profile solution. Taken together, we concluded that the five- and four-profile models were over-extracted, given that too small a percentage of the total sample was assigned to at least one profile that exhibited only minor differences in response patterns when compared to another profile (**Supplementary Figure 1**). Next, the information criterion indices and BLRT suggested that the three-profile solution fit better than the two-profile solution; however, the LMR did not indicate that the three-profile solution was superior. To adjudicate these findings, we followed Nylund, Asparouhov, and Muthen’s recommendation to stop increasing the number of classes when the LMR becomes non-significant, given that the true solution can still be identified in a model with an extra profile but not in a model containing too few profiles (30). Entropy and average posterior probability values also suggested high classification accuracy for the three-profile solution; thus, participants were a good fit for the profile to which they were assigned and not any of the remaining profiles. In summary, we determined the three-profile solution to be optimal.

**Table 2:** Fit indices for latent profile analysis using moral injury and distress scale part 2 items.

Model	AIC	BIC	aBIC	LRT	BLRT	Entropy	APPs	Number of profiles with < 5% of cases
1-Profile	31469.07	31629.96	31515.67	–	–	–	–	0
2-Profile	27085.94	27331.74	27157.12	p <.001	p <.001	.987	.992-.997	0
3-Profile*	**25801.19**	**26131.92**	**25896.97**	**p = .113**	**p <.001**	**.977**	**.978-.993**	**0**
4-Profile	25348.33	25763.97	25468.70	p = .593	p <.001	.959	.936-1.00	1
5-Profile	25025.15	25525.70	25170.11	p = .219	p <.001	.969	.937-.997	2

AIC, Akaike information criterion; BIC, Bayesian information criterion; aBIC, sample-size adjusted Bayesian information criterion; LRT, Lo-Mendel-Rubin likelihood ratio test; BLRT, Bootstrap likelihood ratio test; APPs, Average Posterior Probabilities. *Optimal model appears in bolded text.

### Profile assignment and labelling

**Figure 1:**
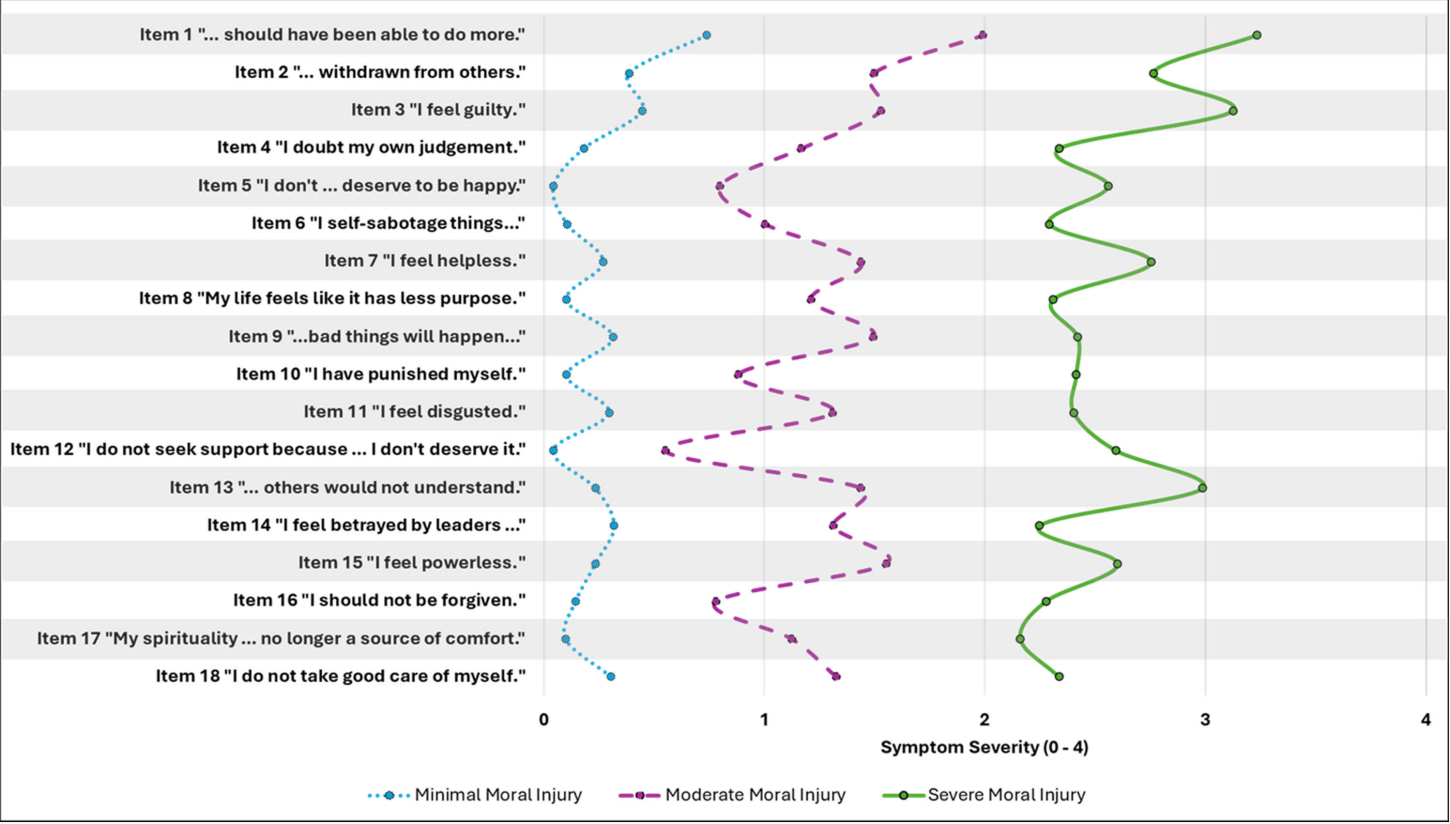
Estimated item-level mean scores for the 3-profile solution indicating severity of moral injury symptoms. Symptom severity scores on the X axis include 0 = not at all, 1 = a little bit, 2 = moderately, 3 = quite a bit, and 4 = extremely. The Moral Injury and Distress Scale was used to assess psychological, emotional, social, and spiritual features of moral injury in a sample of military Veterans, healthcare workers, and first responders exposed to a potentially morally injurious event (*N* = 645).

As illustrated in **Figure 1**, visual inspection of the estimated item-level mean scores for the three-profile solution identified three clinically relevant profiles of moral injury symptoms (**Supplementary Figure 2**). Approximately three out of every four participants (74.7%, *n* = 482) were categorized into a *minimal moral injury profile*, with mean scores on each MIDS item between “not at all” and “a little bit.” About one in five participants (20.2%, *n* = 130) were assigned to a *moderate moral injury profile*, with mean scores on most MIDS items falling between “a little bit” and “moderate” levels of distress. To further characterize this group and provide clinically relevant information, we visually inspected item-level scores and found that average scores were highest on MIDS items 1 (“I think about how I should have been able to do more”) and 3 (“I feel guilty”) for respondents assigned to this profile. Next, one in twenty participants (5.1%, *n* = 33) were assigned to a *severe moral injury profile*, with mean scores indicating greater than moderate levels of distress on each MIDS item. Item-level scores were again highest on items 1 and 3, with participants also reporting elevated symptom severity on item 2 (“withdrawn from others”), item 7 (“feel helpless”), and item 13 (“not seek support because others would not understand”).

**Figure 2:**
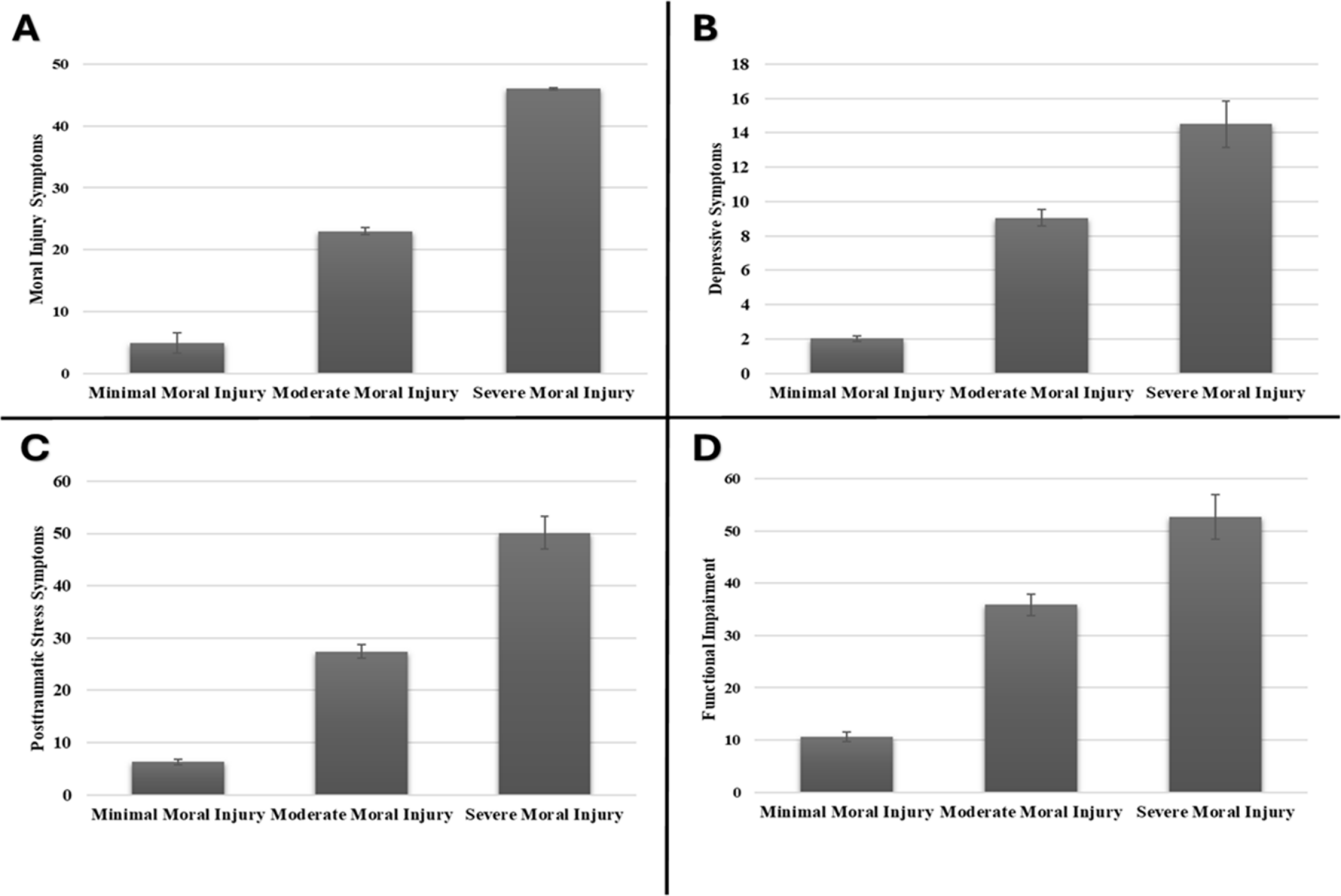
Differences in mental health symptom severity on measures of moral injury **(A)**, depression **(B)**, posttraumatic stress **(C)**, and functional impairment **(D)** for participants exhibiting minimal, moderate, and severe moral injury. Scales used included the Moral Injury and Distress Scale to assess moral injury symptoms, Patient Health Questionnaire to assess depressive symptoms, Posttraumatic Stress Disorder Checklist – 5 to assess posttraumatic stress symptoms, and Brief Inventory of Psychosocial Functioning to assess functional impairment.

We then compared MIDS total scores between the profiles and found that participants assigned to the severe moral injury profile exhibited the highest levels of morally injurious psychological, emotional, social, and spiritual sequelae (*M* = 45.99, *SE* = 1.65, Range = 33.00-68.00; see **Figure 2A**), followed by those assigned to the moderate moral injury profile (*M* = 22.99, *SE* = 0.58, Range = 11.00-41.00; *χ*^2^ = 174.05, df = 1, *p* <.001), and finally those assigned to the minimal moral injury profile (*M* = 4.90, *SE* = 0.19, Range = 0.00-16.00; *χ*^2^ = 609.53, df = 1, *p* <.001). Participants assigned to the moderate moral injury profile also reported greater levels of morally injurious sequelae than those assigned to the minimal moral injury profile (*χ*^2^ = 894.20, df = 1, *p* <.001). Using the established cut score on the MIDS for identifying probable moral injury, 100.0% of those assigned to the severe moral injury profile screened positive on the MIDS, followed by 25.4% of those assigned to the moderate moral injury profile, and none of those assigned to the minimal moral injury profile.

### Mental health outcomes by profile of moral injury symptoms

We next compared mean levels of mental health symptom severity between the profiles. Participants assigned to the severe moral injury profile reported the highest level of depression (*M* = 14.51, *SE* = 1.34, see **Figure 2B**) and posttraumatic stress symptoms (*M* = 50.12, *SE* = 3.15, see **Figure 2C**). By comparison, participants assigned to the moderate moral injury profile reported significantly less severe depression (*M* = 9.05, *SE* = 0.49; *χ*^2^ = 14.50, df = 1, *p* <.001) and posttraumatic stress symptoms (*M* = 27.43, *SE* = 1.29; *χ*^2^ = 43.72, df = 1, *p* <.001). Those assigned to the minimal moral injury profile reported the least severe depression (*M* = 2.03, *SE* = 0.17) and posttraumatic stress (*M* = 6.31, *SE* = 0.50), relative to those assigned to the severe moral injury profile (*χ*^2^*^PHQ^* = 85.00, df_PHQ_ = 1, *p*_PHQ_ <.001; *χ*^2^*^PCL^* = 188.36, df_PCL_ = 1, *p*_PCL_ <.001) and those assigned to the moderate moral injury profile(*χ*^2^*^PHQ^* = 175.71, df_PHQ_ = 1, *p*_PHQ_ <.001; *χ*^2^*^PCL^* = 225.43, df_PCL_ = 1, *p*_PCL_ <.001).

Findings on a measure of functional impairment were similar. Participants assigned to the severe moral injury profile endorsed greater impairment (*M* = 52.73, *SE* = 4.25, see **Figure 2D**) compared to those assigned to the moderate moral injury profile (*M* = 35.86, *SE* = 2.06; *χ*^2^ = 12.66, df = 1, *p* <.001) and those assigned to the minimal moral injury profile (*M* = 10.62, *SE* = 0.91; *χ*^2^ = 94.04, df = 1, *p* <.001). Those assigned to the moderate moral injury profile also reported worse impairment relative to those assigned to the minimal moral injury profile (*χ*^2^ = 105.37, df = 1, *p* <.001).

Over two-thirds of those assigned to the severe moral injury profile screened positive for probable depression (75.8%) or posttraumatic stress (84.8%), and about one-third of those assigned to the moderate moral injury profile screened positive for probable depression (35.4%) or posttraumatic stress (27.7%). In contrast, none of those assigned to the minimal moral injury profile scored higher than the threshold for moral injury, and very few screened positive for probable depression (4.3%) or posttraumatic stress (3.5%). Furthermore, 78.8% of those assigned to the severe moral injury profile screened positive for at least a moderate level of functional impairment, compared to 50.0% of those assigned to the moderate moral injury profile, and 12.2% of those assigned to the minimal moral injury profile.

### Risk correlates of assignment to moderate and severe moral distress profiles

Finally, we examined correlates of assignment to the moderate and severe moral injury profiles compared to the minimal moral injury profile (**Table 3**). After accounting for all other variables in the model, Hispanic respondents of any race (n = 62) had a predicted probability (PP) of 0.468 (SE = .011) for being assigned to the moderate or severe moral injury profile versus the minimal moral injury profile. White, non-Hispanic respondents (n = 499) had a predicted probability of .220 (SE = 003), corresponding to a significantly elevated odds of assignment to the moderate/severe moral injury profiles for Hispanic respondents (adjusted odds ratio [AOR] = 2.96, 95% confidence interval [CI] = 1.70, 5.16). Odds of assignment to the moderate or severe profiles versus the minimal profile did not differ between White, Non-Hispanic respondents and non-Hispanic respondents of other races. Each additional year of age was associated with slightly lower odds of assignment to the moderate or severe profile versus the minimal profile (AOR = .987, 95% CI = .974,.999). Relative to respondents with an associate’s degree or fewer years of education (n = 286, PP = .283, SE = .005), those with a master’s degree or higher (n = 158, PP = .190, SE = .006) also had lower odds of assignment to the moderate or severe profiles (AOR = .56, 95% CI = .34,.91). No difference was observed between those with an associate’s degree or fewer years of education and those with a bachelor’s degree. Similarly, odds of assignment to the moderate or severe profiles did not differ by sex or religious affiliation.

**Table 3:** Models predicting moral injury symptom profile membership.

Variable	Estimate	Std. error	Odds ratio	95% Confidence interval
Sex (ref: female)				
Male	-0.03	0.20	0.97	0.65 - 1.45
Age				
	**-0.01**	**0.01**	**0.99**	**0.97 - 0.99**
Race and ethnicity (ref: White, Non-Hispanic)				
Not White, Non-Hispanic	0.33	0.27	1.39	0.81 - 2.36
**Hispanic**	**1.09**	**0.28**	**2.96**	**1.70 - 5.16**
Education (ref: Assoc. Degree or lower)				
Bachelor’s degree	-0.18	0.22	0.84	0.55 - 1.28
**Master’s degree or higher**	**-0.58**	**0.25**	**0.56**	**0.34 - 0.91**
Religiousness (ref: Atheist/Agnostic)				
Religiously affiliated	0.03	0.31	1.03	0.56 - 1.89
Spiritual but not Religious	0.37	0.27	1.45	0.85 - 2.48

Bolded effects are statistically significant at p <.05. We specified profile membership as the dependent variable, with assignment to the modal profile (minimal symptoms) as the reference category compared to all other profiles. We aggregated symptomatic profiles to reduce the likelihood of biased model parameters for symptomatic profiles (i.e., severe symptoms) containing a small percentage of the overall sample.

## Discussion

The purpose of this study was to identify groups of participants with distinct moral injury symptom presentations by examining response patterns to the Moral Injury and Distress Scale (MIDS) items. Overall, in our population-based sample of military veterans, healthcare workers, and first responders, three out of four individuals did not report a detectable level of moral injury symptoms, suggesting that these individuals never experienced any moral injury symptoms despite being exposed to a PMIE or their symptoms were acute and resolved by the time of the survey. This finding aligns with research on other stress-related syndromes, where most individuals exposed to highly stressful or potentially traumatic events do not experience significant symptoms and resilience is the norm (35).

However, one in 20 participants (5.1%) reported severe moral injury symptoms. These individuals reported a range of clinical features, including intractable moral emotions like guilt (36); social withdrawal (37); and a loss of meaning or purpose in life (38) – all of which are known risk factors for suicidal thoughts and behaviors (39–41). An additional one in five participants (20.2%) reported moderate levels of moral injury, which was characterized by similar symptoms of guilt and hindsight bias but with potentially less emphasis on perceived rejection by close others or valued groups and feelings of powerless related to the perceived inevitability of transgression or impossibility of repair. These individuals may also benefit from clinical interventions that target moral injury or ongoing monitoring, depending on symptom progression and associated impairment. Importantly, before the development of tools like the MIDS, there was no way to efficiently screen individuals for moral injury symptoms and determine who may benefit from targeted treatment. The MIDS fills this gap by measuring PMIE exposure, and the psychological, emotional, social, and spiritual impacts of PMIE exposure.

The MIDS also detects distress not accounted for by other commonly used screening measures like the PCL-5 (22) and PHQ-9 (25), which respectively measure posttraumatic stress and depression symptom severity. While all participants in the severe moral injury profile screened positive for probable moral injury on the MIDS, scores on measures of posttraumatic stress and depression were within normal limits for about one in ten of these participants (9.1%). Without including the MIDS in an assessment battery, these individuals may not be flagged for evaluation and treatment. This would be a missed opportunity given that screening positive for probable moral injury on the MIDS has been associated increased risk of adverse outcomes, including 3-to-6-fold elevated odds of suicidal ideation and attempt (42). Taken together, these findings suggest that individuals in high-stress, service-oriented occupations seeking care for PTSD and depression might also be evaluated and treated for moral injury, especially in cases where residual symptoms persist following delivery of frontline mental health treatments. Also, future research is needed to further explore areas of overlap and distinction between moral injury and common mental health comorbidities, especially across different samples given that rates of exposure to specific types of occupational hazards (e.g., situations involving risk of serious injury or death) vary across groups included in our sample.

Finally, those at particularly elevated risk of assignment to the moderate and severe moral injury symptom presentations included Hispanic individuals of any race versus White, Non-Hispanic individuals, younger versus older individuals, and individuals with fewer versus more years of education. These results highlight the need for more research on moral injury in racial and ethnic minority groups, who may have distinct moral frameworks or trauma histories that heighten vulnerability to moral injury (43). We also replicated prior findings showing that younger individuals report more severe moral injury symptoms than older individuals (44), and fewer years of education emerged as a risk factor in our sample. Further research is needed to explicate these differences, potentially indicating that age-related changes or years of education could prepare individuals to negotiate overlapping and potentially contradictory moral principles (45). Related, future research could broaden its scope beyond individual-level characteristics to examine other factors associated with risk of severe moral injury symptoms including features of PMIE exposure itself—for example, whether the person committed, failed to prevent, or witnessed a morally injurious act. We did not pursue this question in the current paper because the sample was limited to only those who endorsed PMIE exposure, which excluded individuals who denied PMIE exposure as a referent for comparison.

## Limitations

These findings should be considered in light of three key limitations. First, while our population-based sample is more generalizable to community-based samples of military veterans, healthcare workers, and first responders, findings may differ in treatment-seeking samples. In treatment-seeking samples a higher percentage of the sample is likely to screen positive for probable moral injury, with more individuals falling into the moderate and severe profiles. This may also yield greater variability in clinical presentations of moral injury symptoms [e.g., internalizing and externalizing presentations (46, 47)]. Similarly, we aggregated groups that comprised a small percentage of our overall sample to facilitate analysis, which might conceal meaningful differences between groups. Future studies may oversample these individuals to examine differences between underrepresented groups, such as those belonging to minority racial and religious groups and the various occupations included among first responders (e.g., fire service, law enforcement service, etc.).

Second, all data were collected using self-report questionnaires, which are vulnerable to recall and social desirability biases (48). Future studies would benefit from multi-method approaches, including interviews and clinician assessments, as well as data collection immediately following when the event occurred. Notably, however, there is no clinician-administered interview specifically designed to assess moral injury severity—similar to the Clinician-Administered PTSD Scale for DSM-5 (CAPS-5; (49). Finally, the cross-sectional nature of the data limits our ability to draw conclusions about changes in moral injury symptom profiles over time. As longitudinal studies using new moral injury measures become available, person-centered methodologies (e.g., latent growth modeling) may help identify trajectories of moral injury symptoms.

## Future directions for clinical research

Several psychotherapeutic treatments that address moral injury are supported by at least one randomized clinical trial (RCT) or pilot trial: Adaptive Disclosure [AD; (50)], Building Spiritual Strength [BSS; (51)], Impact of Killing [IOK; (52)], and Trauma-Informed Guilt Reduction therapy [TrIGR; (53)]. However, all of these trials were conducted before the development of scales designed to assess the symptoms of moral injury including the MIDS. Thus, eligibility decisions in these trials have depended on patients screening positive for a proxy for moral injury, most commonly PTSD. Unfortunately, to the extent that PTSD and moral injury are distinct syndromes, use of proxy measures as an eligibility criterion likely prevents some individuals from accessing needed treatments (e.g., if a person would have screened positive for moral injury when assessed with the MIDS but screened negative on a proxy like PTSD and was found ineligible). Effectiveness evaluations may also be diminished if change over the course of targeted moral injury treatment is assessed using proxy measures, especially considering that PTSD symptom severity focuses on biobehavioral fear responses that can differ substantially from moral injury symptoms. Taken together, identification of distinct moral injury clinical presentations may help to identify who needs treatment, what kind of treatment they need, and how to evaluate clinical response. It is essential that future clinical trials testing treatments theorized to support recovery from moral injury use scales specifically designed for assessing the psychological, emotional, social, and spiritual features of moral injury. The MIDS appears uniquely well-suited for use in RCTs, given that total scores equal to or greater than 27 indicate probable moral injury, which may be used as an eligibility criterion, and continuous total scores may be examined to assess change over the course of treatment.

## Conclusion

In this population-based sample of veterans, healthcare professionals, and first responders who completed the Moral Injury and Distress Scale (MIDS), we identified three profiles – minimal, moderate, and severe moral injury symptoms. Although most participants reported minimal moral injury, one-quarter endorsed moderate (20.2%) to severe (5.1%) levels of moral injury symptom severity, thus highlighting the burden of moral injury in this population. Results of this study highlight the clinical utility of the MIDS as an effective tool to identify those experiencing severe levels of moral injury and those reporting subclinical levels of moral distress. Its use in measurement-based care may improve detection and treatment planning—ultimately expanding access to emerging interventions for moral injury across diverse care settings.

## Data Availability

The raw data supporting the conclusions of this article will be made available by the authors, without undue reservation.
