# Supplementary figures and images for: The role of measurement in moral injury care: latent profiles on the moral injury and distress scale
Source: Front Psychiatry. 2026 Jan 19;16:1691018. doi: 10.3389/fpsyt.2025.1691018 (PMC12861880; doi:10.3389/fpsyt.2025.1691018)

**Supplemental Figure 1.** Estimated means by profile for solutions with 1-5 profiles.

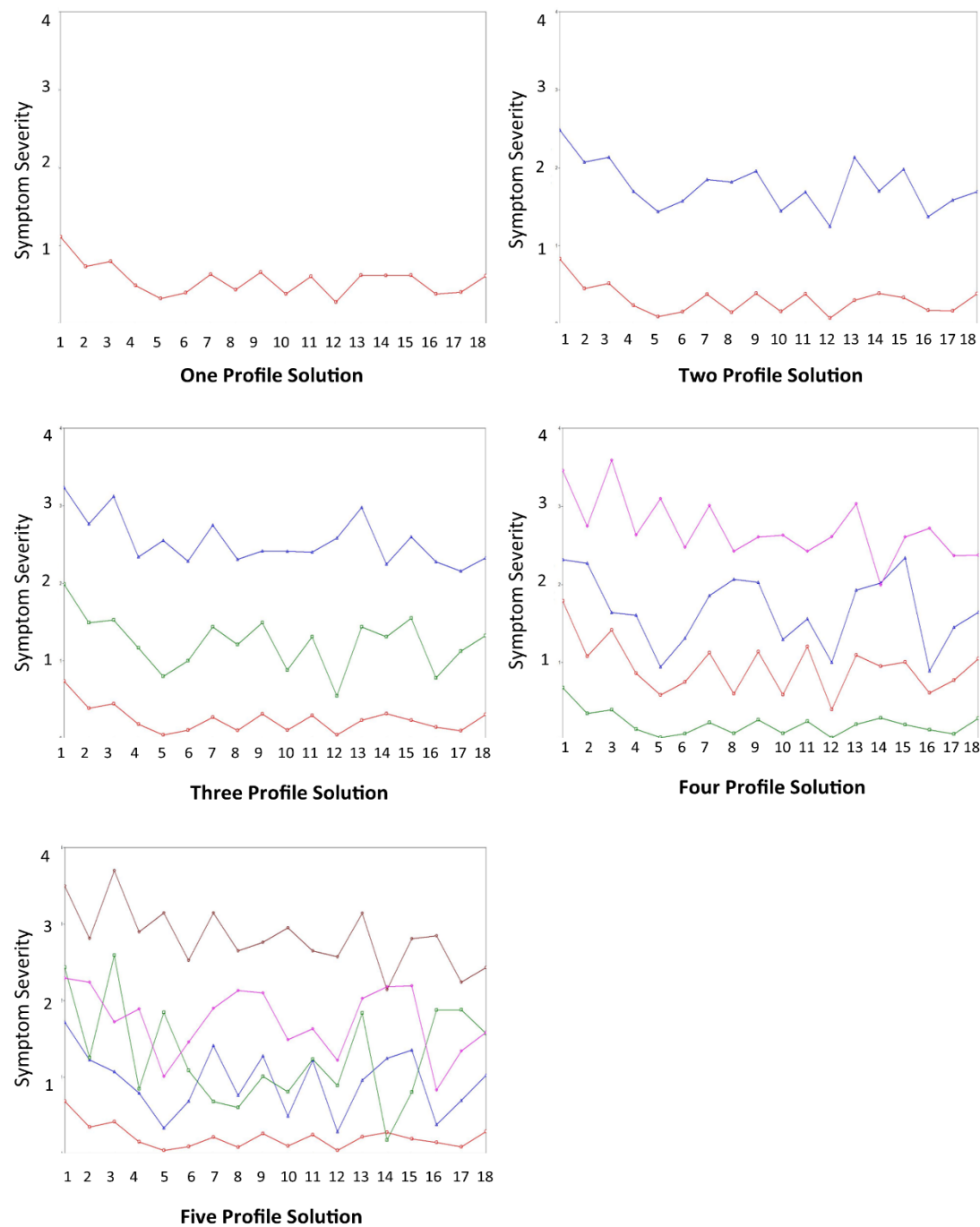

Supplement: Supplementary file 1 [file Image1.pdf]

**Supplemental Figure 2.** Estimated means and observed trajectories for 3-profile solution.

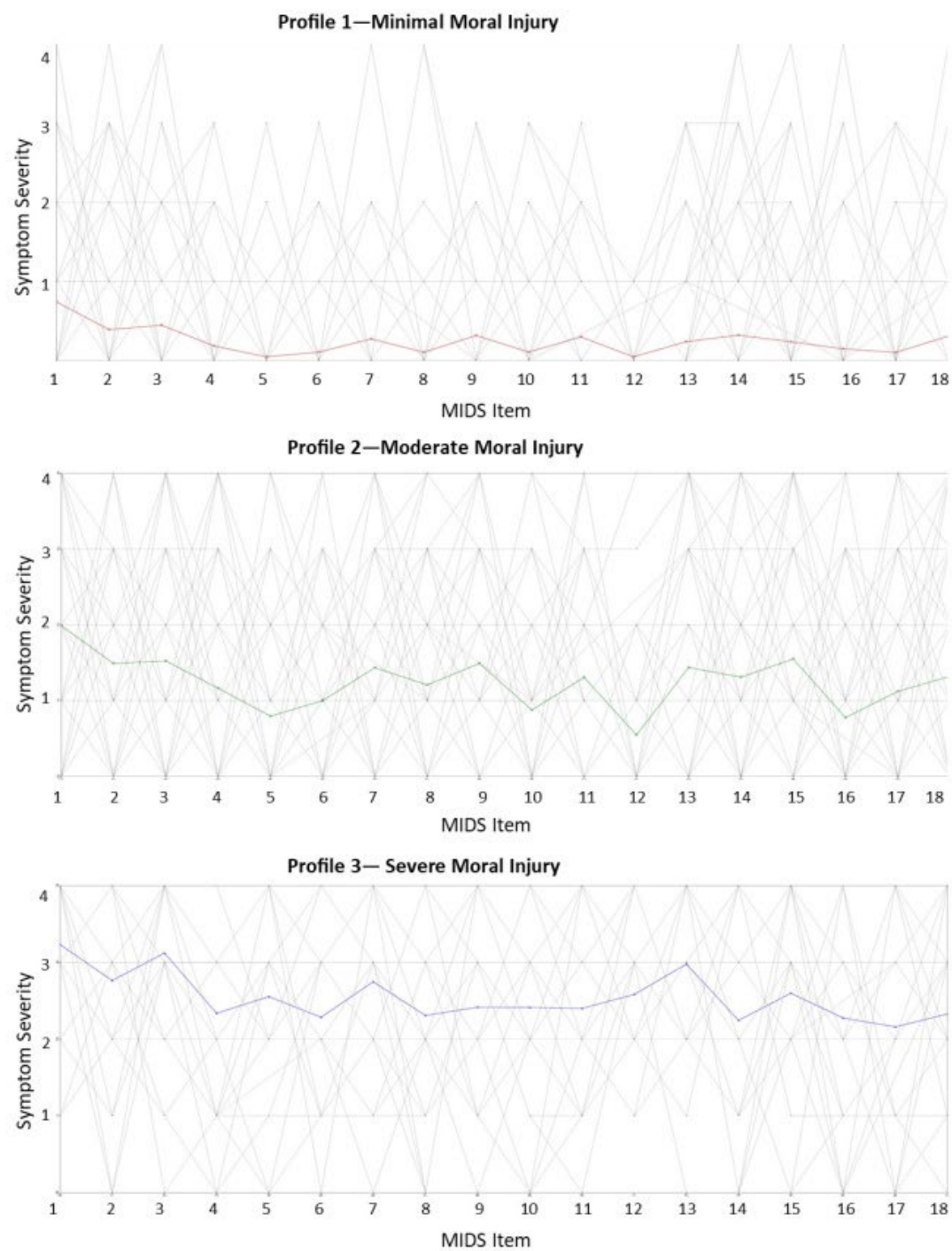

Supplement: Supplementary file 2 [file Image2.pdf]
